# Functional fermented fruit juice production and characterization by using water kefir grains

**DOI:** 10.1007/s13197-025-06209-y

**Published:** 2025-01-29

**Authors:** Dilara Nur Dikmetas, Emine Gizem Acar, Fatma Duygu Ceylan, Furkan İlkadım, Hayrettin Özer, Funda Karbancioglu-Guler

**Affiliations:** 1 Department of Food Engineering, Faculty of Chemical and Metallurgical Engineering, Istanbul Technical University, Maslak,, Istanbul 34469 Türkiye; 2 Tubitak MAM Food Institue/Tubitak MAM, Kocaeli, Türkiye

**Keywords:** Plant-based beverages, Fermentation, Fruit juices, Functional beverage, Yeast, Lactic acid bacteria

## Abstract

In this study, production and characterization of probiotic beverages using water kefir grains with apple, orange, dragon and kiwifruit juices were aimed. The total phenolic and antioxidant capacity, phenolic profiles and physicochemical properties of the beverages were investigated to determine the impact of fermentation. The results showed that fermentation period significantly affects the lactic acid bacteria and yeast counts, organic acid content and phenolic profile of the produced beverages. The yeast, mesophilic lactic cocci and *Lactobacillus* count in beverages were changed within the range 5.59–8.57 log CFU/mL, 4.20–7.49 log CFU/mL and 4.36–7.37 log CFU/mL, respectively. The highest *Lactobacillus* spp. and mesophilic lactic cocci counts were determined in dragon juice (7.78 log CFU/mL) and kiwifruit juice (7.23 log CFU/mL) (48 h), respectively. Up to 21 phenolic compounds were identified in the juice samples. Chlorogenic acid and rutin were the major phenolic compounds detected in all fruit juices samples. Lactic acid level in all beverages increased during fermentation up to 9.03 g/L in fermented dragon juice. Furthermore, the ethyl alcohol concentration in beverages is within the regulatory limitations for non-alcoholic beverages. According to the finding, dragon, apple, orange and kiwifruit juices can be fermented with water kefir grains to produce non-dairy, functional, and probiotic beverages.

**Graphical Abstract:**

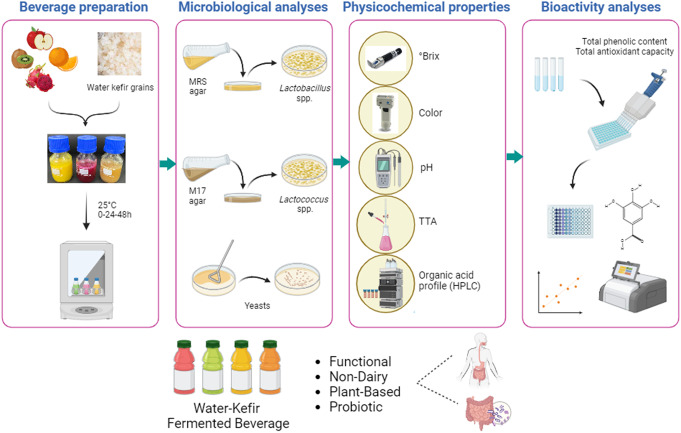

**Supplementary Information:**

The online version contains supplementary material available at 10.1007/s13197-025-06209-y.

## Introduction

Fermentation technology and the popularity of fermented products have risen in recent years because consumers’ demand tends toward more natural, chemical-free, clean, functional, and sustainable food, with increased preferences for being vegan, vegetarian, and flexitarian. Moreover, it is now more requested to consume healthy and immune-enhancing bioactives from foods and essential nutrients (Corona et al. 2016). When all these situations are evaluated together, it is not surprising that animal-based foods have decreased dramatically, and the tendency toward plant-based foods has increased. It has been reported that the 3-year dollar growth of plant-based foods will be 44% in 2022 (Good Food Institute, 2022).

To replace or reduce the consumption of animal-based foods and ingredients from the diet, fruits and vegetables, cereals, nuts, and seeds have become the most commonly used commodities (Alves et al. 2021). To diminish postharvest losses and increase product diversity in the market, fruits, which are frequently recommended for consumption because they are effective against various diseases, are processed in both traditional and innovative ways (Randazzo et al. 2016). While fermentation preserves nutritional properties and provides health benefits because it does not require heat (Araújo et al. 2023), using fruits to make fermented beverages is one of the strategies to minimize losses and serve new products to consumers (Bueno et al. 2021).

The industry collaborates with researchers to create innovative technologies and food products that cater to specific requirements and offer advantages, such as functional meals and nutraceuticals. Functional foods are those that, upon consumption, produce nutritional and physiological impacts on the body (da Silva Gomes et al. 2024). Probiotics are defined as “live microorganisms which, when administered in adequate amounts, confer a health benefit on the host” by the Food and Agriculture Organization/World Health Organization (FAO/WHO). Probiotics and their therapeutic effects in in managing, preventing, and treating several diseases have gained significant attention for many years. Consequently, the need for probiotic food and pharmaceutical products is growing day by day (Homayouni Rad et al. 2023). Moreover, the evidence suggests that probiotic intake in infants may protect against prevalent respiratory tract illnesses (Rad et al. 2013b). Probiotics are susceptible to environmental stresses. Manufacturing activities like heating, probiotic metabolites generated during storage, including acids, and gastrointestinal circumstances such as gastric acid and bile salts jeopardize the survival of these microbes (Rad et al. 2012). Dairy-based, fruit and vegetable-based, legume-based, cereal-based, coffee-based, or tea-based are some categories for functional beverages. Different food matrices like ice cream (Homayouni Rad et al. 2023); yoghurt like fermented beverage (da Silva Gomes et al. 2024) and cereal based baked food (Mani-Lo ´pez et al. 2023) have been studied as ideal delivery vehicles for viable probiotics.

Since dairy products have unique physicochemical and nutritional qualities that offer a buffering capacity for the survival of a viable number of probiotics (6.0–8.0 log CFU/mL) in the harsh acidic conditions of the stomach (pH 2–3), they are regarded as a preferred food matrix for the development of probiotic beverages. Because lactose intolerance is a metabolic disorder associated with obesity, it can disrupt the balance of gut microbiota and baseline ratios of normal flora, which can occasionally limit consumers’ ability to take dairy-based probiotics. For these reasons, dairy-based probiotics may challenge for those with lactose sensitivity and obesity (Meenu et al. 2024). From this point of view, plant-based products with probiotics were developed including have been studied as fruit juice (Ghafari and Ansari 2018; Marius et al. 2023), ice teas formulated with black teas and grape juice (Gülhan 2024), plant-based beverages by fermenting soy, almond, and coconut beverages (Gungor et al. 2024), grain based beverages (Andrade and Casto, 2023),

Fruits have attracted considerable interest in creating probiotic beverages owing to their appealing flavor and widespread acceptance as nutritious and invigorating drinks across all age demographics. Due to its rich nutrient content, including vitamins, minerals, dietary fibers, and antioxidants, the regulated alteration of the fruit juice matrix might render it an optimal substrate for the cultivation of probiotic microorganisms.

Fruit juices pose a great environment to microorganisms, so probiotic growth and viability, due to their high nutritious content including water, sugar, vitamins, minerals, proteins, enzymes, antioxidants, polyphenols, etc., and also, fermentation increases the already high nutritional value of the fruit juice (Randazzo et al. 2016). Furthermore, lactic acid fermentation of fruits leads to particular flavor and nutrient formation. Thus, new beverages are obtained with unique physicochemical and sensorial characteristics (Mendes et al. 2021). In addition, recent studies have shown that it is possible to enrich selenium in fruit juices by lactic acid fermentation (Paredes et al. 2022).

Non-dairy kefir is produced by fermenting water containing sugar or fruit extract with water kefir grains consisting of lactic acid bacteria (LAB) and yeasts (Randazzo et al. 2016). Its fermentation generally occurs at room temperature for 2–4 days. With the help of its microbial content, fermenting with water kefir produces lactic acid, acetic acid, ethanol, CO_2_, aromatic compounds, etc. (Alves et al. 2021). Because it has an acidic, sour, carbonated, and fruity taste (Moretti et al., 2022), to make kefir more attractive to consumers without any food additives, it is a good way to use fruit juice in fermentation. Unlike milk kefir, water kefir can use a wider range of substrates (Bueno et al. 2021), it ferments sucrose rather than lactose, and it does not leave residual milk products, especially lactose intolerant (Alves et al. 2021), which is important for allergic people or vegan consumers.

Although the content may differ according to the substrate; sugars, organic acids, alcohols, and esters are frequently present in fermented foods and beverages. Because functional foods or beverages decrease the possibility of health problems by increasing the body functions of consumers, these fermented beverages and kefir are also functional beverages because of their probiotic content (Mendes et al. 2021). Water kefir has antimicrobial, antioxidant, anti-inflammatory, immunomodulant, antihypertensive, anti-toxic, anti-ulcerogenic, hypocholesterolemic, hyperglycemic, anti-hyperlipidic, healing, bioactive properties, and probiotic activities (Bueno et al. 2021). In contrast to fermentation with a single strain or a limited selection of strains, fermentation with kefir grain has enhanced potential for producing novel bioactive compounds, due to its extensive microbial variety, ease of storage, and superior flexibility (Du et al. 2023). Moreover, probiotic viability has also been changed according to the type of raw material and food products. From this point of view, limited research has examined the characterization of different types of water kefir fermented fruit juices from several points of view, such as phenolic content, probiotic viability, organic acid, and alcohol content. In light of all this information, the present study aimed to ferment fruit juices using water kefir, thus producing a dairy-free, vegan, and functional beverage with high nutritional value, antioxidant, and phenolic content. In this scope, a healthy alternative will be produced for consumers who do not prefer to use animal-based beverages or who are allergic or lactose intolerant to dairy products, and it will contribute to sustainability and reduce carbon emissions by providing a novel plant-based beverage. For this purpose, four different fruit juices (orange, apple, pitaya, kiwifruit) were used as substrates for water kefir grains, and the physicochemical properties of the functional beverage obtained during two different fermentation periods were investigated.

The objective of this study was to investigate the impacts of water kefir fermentation on the probiotic viability, physicochemical properties, phenolics, antioxidant activity, organic acid and alcohol content of apple, orange, dragon and kiwifruit juices.

## Materıals and method

### Sample and fermented fruit juice preparation

Orange, apple, dragon fruit, kiwifruit, and brown sugar were obtained from a local market in Istanbul, Turkey. Water kefir grains were provided by Danem Inc. (Isparta, Turkey). In a study by Gökırmaklı and Güzel-Seydim (2022), it was reported that water kefir grains include *Bifidobacterium* spp. and *Lactobacillus* spp. *Lactococcus* spp., *Streptococcus* spp. *Acetobacter* spp., and *Gluconobacter* spp. Water kefir grains were kept in a 5% sterile brown sugar solution in a shaking incubator at 25 °C. To maintain water kefir grains viability, the grains were taken into fresh brown sugar solution every 24 h (Bueno et al. 2021).

The fruits were cleaned with tap water, and juices were obtained using an industrial fruit juicer. Fruit juices (FJ) were pasteurized for 5 min at 75° C, then cooled to room temperature (Randazzo et al. 2016). Kefir grains (%1, w/w) were inoculated into the obtained fruit juices and fermentation was statically performed at 25 °C for 24 and 48 h. Fermentation was observed with pH control, and subsequent to the incubation period, the physicochemical characteristics and microbiological analyses of the samples were performed. Periodic sampling was used to evaluate the fermentation efficiency and for further analysis. Before the analysis, kefir grains were removed from the fruit juices. Fruit juices were freeze-dried for bioactivity and organic acid analyzes.

### Microbiological analyses

Fermented fruit juices, collected at different fermentation periods (0, 24 and 48 h), and total yeast, rod and coccus LAB counts were determined. Total yeast count was determined on yeast extract peptone dextrose agar (YEPDA) using the spread plate method after aerobic incubation at 25 °C for 48 h. Coccus LAB on M17 agar and rod LAB on Man-Rogosa-Sharpe (MRS) agar were counted using the pour plate method after anaerobic incubation at 37 °C for 48 h. All media and supplements were purchased from Merck. Analyses were conducted with three replicates.

### Determination of the physicochemical properties

The pH of the samples was determined using a digital pH meter (Hanna, HI 9321). The soluble solid content (SSC) was measured using a digital portable refractometer and reported as °Brix. The titratable acidity of the juices was determined according to the method reported by the AOAC (2000), and are expressed using the citric acid conversion coefficient. The color parameters of the fruit juices, where L* is the lightness, a* and b* are color-opponent dimensions, redness, and yellowness, were determined using a hand-held Minolta Chroma Meter Model CR-400 (Minolta Co Ltd., Osaka, Japan). The color change of the fruit juices during the fermentation period was also calculated using the following formula:

1 $$\:{\Delta\:}\mathrm{E}={\left({\left(\mathrm{L}\mathrm{*}-\mathrm{L}0\mathrm{*}\right)}^{2}\:+\:{\left(\mathrm{a}\mathrm{*}-\mathrm{a}0\mathrm{*}\right)}^{2}+\:{\left(\mathrm{b}\mathrm{*}-\mathrm{b}0\mathrm{*}\right)}^{2}\right)}^{\mathrm{0,5}}$$

### Chromatographic analysis of sugars, organic acids, and alcohols

The concentrations of oxalic, tartaric, malic, lactic, acetic, citric, fumaric, quinic, and ascorbic acids were determined in all fruit juice samples at 0,24 and 48 h by (HPLC) (Agilent Technologies Co., Ltd, 1100 series, Germany) equipped with a quaternary pump and a diode array detector (DAD, Agilent series 1200, Agilent Technologies Co., Ltd, Germany)(Gao et al. 2022). Before injection, all samples were filtered using PDFE 0.22 μm. With an isocratic elution ratio of 97.5:2.5 (v/v) for 30 min, the mobile phase was formed of solvent A (water containing 0.1% phosphoric acid) and solvent B (methanol). The flow rate was 0.5 mL/min at 210 nm. The measurement of organic acids results in the construction of calibration curves for each constituent using pure standards at various concentrations. Juices ethyl alcohol content were analysed using a method modified by the AOAC 983.13 technique by gas chromatography (Thermo Trace GC, Milan, Italy).

### Determination of the total phenolic content

The total phenolic content was calculated using the Folin-Ciocalteu reagent. 100 µL of samples were mixed with 0.75 mL of 0.2 N Folin-Ciocalteu reagent (10%). The mixture was then mixed with 750 µL of saturated Na_2_CO_3_ (6%) solution after the samples were held in the dark for 5 min. A UV-vis spectrophotometer (BioTek Instruments, Winooski, Vermont, USA) was used to measure the absorbance of the samples at 765 nm after shaking and leaving them at room temperature in the dark for 90 min. Three replicates of each sample were used to prepare it. The results were presented as mg gallic acid equivalent/100 mg sample.

### Determination of total antioxidant capacity

The total antioxidant capacity of the samples was evaluated using two different methods: the 2,2-diphenyl-1-picrylhydrazyl (DPPH) assay and the cupric ion-reducing antioxidant capacity (CUPRAC) assay. Two milliliters of a 0.1 mM DPPH radical reagent were added to 100 µL of samples. After that, the samples were held in the dark for 30 min. The absorbance was then measured at 517 nm in a UV-vis spectrophotometer (BioTek Instruments, Winooski, Vermont, USA). CUPRAC was determined according to the method of Apak (2004). Briefly, 100 µL of samples were mixed with 1 mL distilled water, 1 mL 10 mM CuCl_2_, 1 mL 7.5 mM neocuprine, and 1 mL ammonium acetate (NH_4_Ac) (pH 7) buffer solution and shaken. After 30 min at room temperature, the absorbance of the samples was measured at 450 nm using a UV-vis spectrophotometer (BioTek Instruments, Winooski, Vermont, USA).

### Determination of phenolic profiles by HPLC

A HPLC system was used to evaluate the profiles of phenolic compounds derived from fermented fruit juice samples (Capanoglu et al. 2008). A Waters 2695 HPLC system with PDA dedector (Waters 2996) was used to analyze juice samples after filtering them through a 0.45 μm membrane filter. The column used was a Supelcosil LC-18 (25 cm × 4.60 mm, 5 m column Sigma-Aldrich, Steinheim, Germany). As part of the mobile phase, the following solvents were used: solvent A, Milli-Q water containing 0.1% of (v/v) trifluoroacetic acid (TFA), and solvent B, acetonitrile containing 0.1% of (v/v) TFA. There was a linear gradient apllied as follows: 95% solvent A and 5% solvent B at 0 min; 65% solvent A and 35% solvent B at 45 min; 25% solvent A and 75% solvent B at 47 min and at 50 min returning to initial conditions. The flow rate was 1 mL/min. The detection wavelengths were 280, 312, 360 and 520 nm. A characteristic UV spectrum and retention times were used to identify the phenolic compounds. A set of external standards was used for the quantification.

### Statistical analyses

Statistical analyzes were performed using Minitab (Ver. 18.0, USA). A one-way analysis of variance (ANOVA) was conducted to determine the statistically significant difference, followed by Tukey’s multiple comparison test.

## Results

### Microbial growth dynamics during fermentation

**Fig. 1:**
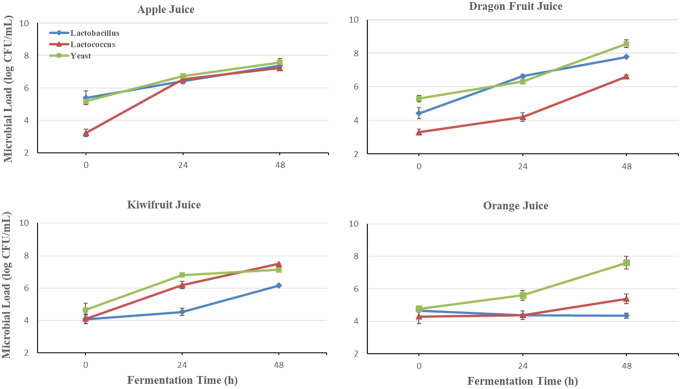
Microbial growth dynamics during the fermentation of fruit juices with water kefir

*Lactobacillus* spp., mesophilic lactic cocci, and yeast loads during the fermentation of apple, dragon, kiwi, and orange juices were determined, and the microbial growth dynamics are shown in Fig. 1. Before inoculating water kefir grains, in pasteurized fruit juices, the count of *Lactobacillus* spp., mesophilic lactic cocci, and yeast were below the detection limit (< 1 log CFU/mL). Kefir is regarded as a probiotic product with several therapeutic advantages (Otles & Cagindi, 2003).

In almost all fruits, at the time of water kefir inoculation (0 h), the highest viability was in yeast (4.65–5.30 log CFU/mL), whereas mesophilic lactic cocci had the lowest log count (3.22–4.29 log CFU/mL). While the highest count of mesophilic lactic cocci was 7.49 ± 0.04 log CFU/mL after 48 h fermentation of kiwifruit juice, this rate was 7.78 ± 0.04 and 8.57 ± 0.18 log CFU/mL for *Lactobacillus* spp. and yeast after 48 h fermentation of dragon fruit juice, respectively. *Lactobacillus* counts were significantly higher in apple and dragon juice than in orange and kiwifruit juices. Additionally, while yeast counts were significantly higher in dragon juices, mesophilic lactic cocci counts were higher in kiwifruit juices at both fermentation times (Table S1) Moreover, yeast and LAB counts showed that orange, apple, kiwifruit, and dragonfruit were potential substrates for water kefir fermentation.

### Physicochemical properties of fermented fruit juices

**Fig. 2:**
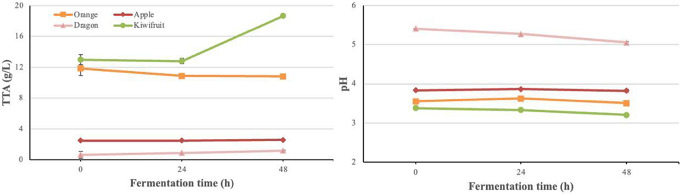
Changes in pH and TTA values of fruit juices during fermentation with water kefir

As physicochemical properties affect the quality of fruit juices, pH and TTA (Fig. 2), TSS and color parameters (Table 1) were measured.

**Table 1:** Changes in the color parameters and TSS content of juices during fermentation with water kefir

	L*	a*	b*		ΔE	TSS (°Brix)
*Apple Juice*						
NF	58.85 ± 2.01^B^	-2.38 ± 0.22^B^	24.42 ± 0.37^B^		-	11.63 ± 0.48
F24	65.44 ± 1.29^A^	-1.90 ± 0.57^B^	22.29 ± 1.97^B^		6.95	11.25 ± 0.29
F48	51.52 ± 1.79^C^	-1.30 ± 0.30^A^	26.63 ± 1.03^A^		7.73	11.00 ± 0.00
*Dragon Fruit Juice*						
NF	32.40 ± 1.45^A^	43.46 ± 2.08^B^	11.79 ± 1.58^B^		-	9.75 ± 0.29
F24	30.80 ± 0.90^A^	43.08 ± 1.05^B^	16.09 ± 0.28^A^		4.59	9.38 ± 0.25
F48	31.98 ± 0.65^A^	47.01 ± 0.52^A^	13.08 ± 0.30^B^		3.79	9.00 ± 0.00
*Kiwifruit Juice*						
NF	39.61 ± 0.61^B^	-5.10 ± 0.05^B^	15.01 ± 1.03^B^		-	12.50 ± 0.07
F24	35.79 ± 1.36^C^	-3.90 ± 0.85^A^	8.35 ± 0.38^C^		7.76	12.50 ± 0.13
F48	46.55 ± 1.43^A^	-3.55 ± 0.22^A^	19.20 ± 0.82^A^		8.26	11.55 ± 0.09
*Orange Juice*						
NF	67.66 ± 2.33^A^	-9.67 ± 0.61^A^	68.77 ± 1.47^A^		-	12.25 ± 0.29
F24	67.87 ± 1.23^A^	-10.23 ± 1.01^A^	68.27 ± 1.73^A^		0.78	12.00 ± 0.25
F48	67.96 ± 0.66^A^	-9.82 ± 0.29^A^	67.92 ± 1.21^A^		0.91	11.88 ± 0.25

^A, B, C^ values within the same column and the same fruit juice with different superscript upper case are significantly different (*p* < 0.05)

At time 0, the pH of different fruit juices varied between 3.38 and 3.84, while dragon juice was 5.41. Although the pH of dragon juice was higher than that of other fruit juices at all times, the greatest decrease in pH was also observed in fermented dragon juice. After 48 h of fermentation, the measured pH values of apple, dragon, kiwi, and orange juices were 3.83, 5.06, 3.21, and 3.51, respectively. Dragon juice with the highest pH had the lowest TTA values than other fruits, as expected. While the acidity of dragon and kiwi juices increased directly with fermentation, it remained constant in apple juice at 24 h and increased at 48 h, and decreased in orange juice during fermentation. According to TTA results, although dragon juice had the highest percentage increase in acidity during fermentation, the highest acidity was 18.69 g citric acid/L in kiwi juice and the lowest acidity was 1.18 g malic acid/L in dragon juice after 48 h of fermentation. TSS amounts were recorded as ˚Brix at 0, 24, and 48 h fermentation for each fruit juice. The fermentation time and amount of TSS showed an inversely proportional relationship in all fruit juices. The highest amount of TSS was measured in orange juice with 12.25 and 11.88 ˚Brix, and the lowest in dragon juice with 9.75 and 9.00 ˚Brix, at 0 and 48 h, respectively. During the 48-h fermentation, the ˚Brix decrease was seen most in dragon juice with 7.7%, followed by kiwi juice with 7.6%. Color change was calculated by comparing the L*, a*, b* color parameters measured during fermentation with the parameters of the non-fermented control juices. Color change was increased with increasing fermentation time in fruit juices except dragon juice. The ΔE value, which expresses the color change, was found largest in the kiwi juice (8.26) and the smallest in orange juice (0.91) after 48 h of fermentation. Color parameters a* and b* were significantly affected by the fermentation time of apple, dragon, and kiwi fruit juices (Table 1).

### Chromatographic analysis of organic acids and alcohol

The organic acid profiles of unfermented and water kefir fermented juices were determined and their changes during fermentation is given in Table 2. While malic, lactic, acetic, citric, fumaric and ascorbic acids were detected in all fruit juices, oxalic acid was found only in dragon juice, while formic, tartaric and quinic acid were not found in any fruit juice. Organic acid amounts did not only decrease or increase during fermentation, but different changes were obtained in different fruits and different times. In 48-h fermented fruit juices, the highest amount of malic and ascorbic acid were detected in apple juice, citric acid in kiwi juice and fumaric acid in dragon juice. Ethanol concentration were significantly higher in all juices after 48 h of fermentation with water kefir grains, either resulting from yeast metabolism or heterolactic fermentation by LAB (Table 2).

**Table 2:** Organic acid and alcohol (g/L) changes during the fermentation of juices with water kefir

	EtOH	Malic	Lactic	Acetic	Citric	Fumaric	Ascorbic	Oxalic	Formic	Tartaric	Quinic
Orange Juice											
C	ND	1.37	2.59	0.35	19.35	0.02	0.14	ND	ND	ND	ND
F24	0.65	1.26	3.14	0.25	26.85	0.02	0.11	ND	ND	ND	ND
F48	5.60	0.97	4.29	0.19	19.72	0.01	0.08	ND	ND	ND	ND
Apple Juice											
C	ND	4.44	0.22	0.10	0.12	0.01	0.17	ND	ND	ND	ND
F24	0.31	4.65	0.44	0.10	0.15	0.01	0.17	ND	ND	ND	ND
F48	0.32	4.08	1.28	1.21	0.14	0.01	0.17	ND	ND	ND	ND
Dragon Juice											
C	ND	3.43	1.30	0.53	0.42	0.13	0.13	0.21	ND	ND	ND
F24	1.77	5.75	2.47	0.62	0.44	0.66	0.03	0.22	ND	ND	ND
F48	3.06	3.59	9.03	0.94	0.55	0.31	0.03	0.22	ND	ND	ND
Kiwi Juice											
C	ND	3.16	0.22	1.34	29.01	0.01	0.09	ND	ND	ND	ND
F24	0.30	3.03	0.26	1.61	28.17	0.01	0.08	ND	ND	ND	ND
F48	2.54	2.17	0.30	1.77	23.80	0.01	0.08	ND	ND	ND	ND

*Abbrevetions*: F, fermented juices; NF, non-fermented fruit juices; ND, not detected

### Total phenolic and antioxidant capacity

**Fig. 3:**
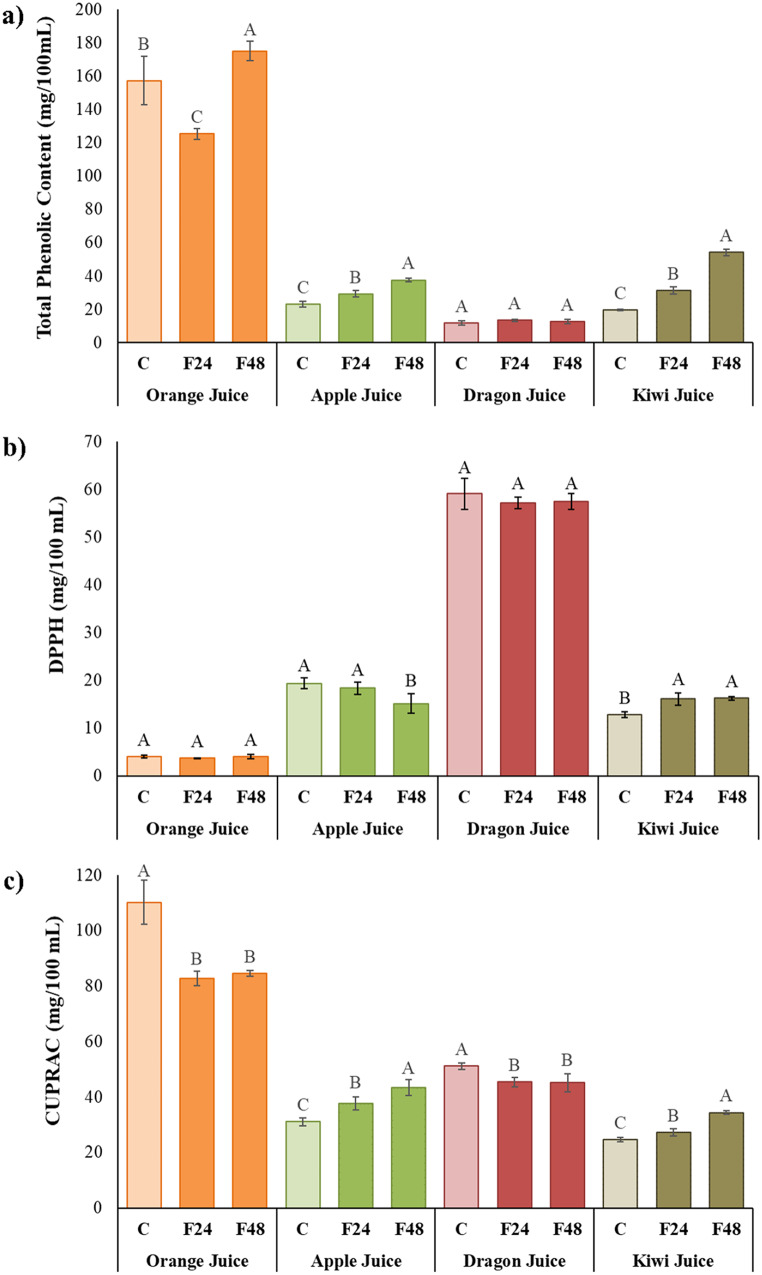
Changes in total phenolic content (**a**:TPC) and total antioxidant capacity (**b**: DPPH and **c**: CUPRAC) of the control and kefir-like juices. **A**, **B**, **C** values within the same fruit juice with different letters are significantly different (*p* < 0.05)

The total phenolic (TP) contents of control and water kefir fermented juices at 24 and 48 h are shown in Fig. 3a. When 48 h amounts were compared, it was observed that fermented juices obtained from fruits, except orange, contained higher amounts of phenolic substances than unfermented fruit juices. However, the highest TP content was measured in unfermented orange juice after 48 h. Considering the 0 and 48 h results, the amount of TP increased in both unfermented and fermented orange and kiwi juices, increased only in fermented apple juice and decreased in both treatments in dragon juice. Total antioxidant activity was examined with both 2,2-diphenyl-1-picrylhydrazyl (DPPH) radical scavenging activity and cupric reducing antioxidant capacity (CUPRAC) assays, and the results are shown in Fig. 3b-c.

### HPLC analysis of phenolic compounds

Fruit juices - orange, apple, dragon fruit and kiwi- were examined for their phenolic composition during fermentation. Changes in phenolic compounds of orange, apple, dragon fruit and kiwi juices during fermentation are shown in Table 3. Up to 21 phenolic compounds were identified in the juice samples. Chlorogenic acid and rutin were the major phenolic compounds detected in all fruit juices samples. The results showed significant changes in the phenolic profiles, emphasizing the complex interaction between fermentation and processes and the phenolic content of the juices (*p* < 0.05).

**Table 3:** Changes in phenolic compounds of orange, apple, dragon fruit, and Kiwi juices during fermentation

Compound/ Sample	Orange Juice			Apple Juice			Dragon Fruit Juice			Kiwi Juice		
	NF	F24	F48	NF	F24	F48	NF	F24	F48	NF	F24	F48
Gallic acid	0.0035	-	-	-	-	-	0.0486^a^	0.1099^c^	0.1767^b^	0.0273^c^	0.0661^b^	0.1074^a^
4-hydroxybenzoic acid	-	-	-	-	-	-	0.0075	-	-	-	-	-
trans-cinnamic acid	-	-	-	-	-	-	-	0.0072	0.0169	-	-	-
Syringic acid	-	-	-	-	-	-	0.4405^a^	0.3051^c^	0.3678^b^	-	-	-
Protocatechuic acid	-	-	-	-	-	-	-	-	-	-	-	0.0245
Catechin	0.1931^a^	0.1715^b^	0.1292^c^	0.0098^a^	0.0063^b^	0.0045^c^	-	-	-	0.0166^c^	0.0229^b^	0.0568^a^
Vanilic acid	-	-	-	-	-	-	-	-	-	-	-	-
Epicatechin	0.7558	-	-	0.0476^a^	0.02^b^	0.0134^c^	-	-	-	0.251^ab^	0.2773^a^	0.2169^c^
Hesperedin	7.1939^ab^	7.2609^a^	7.0361^ab^	-	0.1805	0.3861	-	-	-	-	-	-
Phlorizin	3.0331^b^	5.006^a^	2.597^c^	-	0.4847	0.6641	0.111^b^	0.0805^c^	0.1393^a^	-	-	-
Chlorogenic acid	0.685^c^	0.7898^b^	0.8772^a^	1.1798^c^	3.9118^b^	4.7073^a^	0.0336^c^	0.0549^b^	0.0824^a^	0.6254^a^	0.3276^c^	0.5025^b^
Caffeic acid	0.414^b^	0.2459^c^	0.6434^a^	0.2268^ab^	0.252^a^	0.17^c^	0.1052^b^	0.0711^c^	0.1874^a^	-	-	0.0079
p-coumaric acid	-	-	-	0.1457^c^	0.3087^ab^	0.3746^a^	-	0.053	0.0795	-	-	-
Apigenin	0.5469^a^	0.4528^b^	0.0347^c^	-	-	-	-	-	-	-	-	-
Ferulic acid	-	0.0398	0.0487	-	-	-	0.0077^a^	0.0682^b^	0.0626^bc^	-	-	-
Sinapic acid	-	-	-	-	-	-	0.0442^a^	0.049^a^	0.212^b^	-	-	-
trans resveratrol	-	-	-	0.0636^a^	0.0505^ab^	0.098^c^	-	-	-	-	-	-
Rutin	0.2255^a^	0.1876^b^	0.0884^c^	0.0433^c^	0.0764^b^	0.0846^a^	0.0521	-	-	0.0087^b^	0.0041^c^	0.0101^a^
Quercetin-3-O-galactoside	0.0879^a^	0.0581^b^	0.0068^c^	0.0228^ab^	0.0246^ab^	0.0268^a^	0.1431^ab^	0.1304^ab^	0.17^a^	-	-	-
Quercetin	0.0082^c^	0.0095^b^	0.1747^a^	0.0088^c^	0.0103^b^	0.0173^a^	-	-	-	0.0073^a^	0.0009^c^	0.0057^b^
Kaempherol	0.0039	-	-	-	-	-	-	-	-	-	-	-

*Abbrevetions*: F, fermented juices; NF, non-fermented fruit juices

^a, b, c^ values within the same row and the same fruit juice with different superscript lower case are significantly different (*p* < 0.05

## Discussion

Probiotics are living bacteria that, when administered in sufficient amounts, provide health advantages to the host. Probiotics are supposed to offer various potential health advantages, including the enhancement of the immune system, maintenance of intestinal homeostasis, reduction of cancer risk, improvement of lactose digestion, and prevention of metabolic disorders such as cardiovascular diseases, diabetes, and certain allergic reactions (Meenu et al. 2024). In order to obtain the necessary functionality from the intrinsic microbiota, the product must exhibit minimal cell viability, which, according to the Food and Agriculture Organization of the United Nations/World Health Organization (FAO/WHO, 2002) recommendations, is a minimum of 4 and 7 log CFU/g for yeast and bacteria, respectively (Vimercati et al. 2020). Ensuring the survival of probiotic microorganisms is difficult during the preparation and storage of beverages. The acidic conditions in these medium and low temperatures influence microbe survival rates. The efficacy of probiotic food can be achieved by including the appropriate strain in enough amounts and ensuring the strain’s viability during the product’s shelf life (de Moraes et al., 2024). The microbial growth rates of *Lactobacillus* spp., *Lactococcus* spp. and yeasts found in this study were consistent with the finding that yeasts were dominant, with the researchers’ previous study with kefir fermentation on sugary substrates (Du et al. 2023). It was observed that yeast counts of water kefir beverages after 48 h changed between 7.12 and 8.57 log. Because lactic acid bacteria and yeasts are found in water kefir grains, two different types of fermentation typically occur. Using the homofermentative pathway, lactic acid bacteria turn carbohydrates into lactic acid, and using the heterofermentative pathway, they produce lactic acid, acetic acid, ethyl alcohol, and carbon dioxide (Ozcelik et al. 2021).

Although the growth trends of microorganisms vary depending on the fermentation time and fruit, yeast viability was generally dominant at both fermentation times. For example, in a study conducted by Corona et al. (2016), vegetable kefir-like beverages, rod and coccus LAB contents were similar; however, depending on the substrate, the LAB content changed. In addition, all LAB strains and yeast counts strictly increased during the 24 h of kiwi and apple fermented juice fermentation, reaching a nearly stable period. This phenomenon could be caused by LAB producing large amounts of acid, which inhibits bacterial proliferation while using nutrients to grow and reproduce (Lan et al. 2023). In accordance with the decrease in pH, the acidity of dragon and kiwi juices increased directly with fermentation. Organic acid production in fermented foods lowers pH, increasing total titratable acidity (Puerari et al., 2015). Bueno et al. (2021) also reported a pH reduction in fruit juices fermented with 1% kefir grains from 3.80 to 3.48. Bueno et al. (2021) also reported a pH reduction in fruit juices fermented with 1% kefir grains from 3.80 to 3.48. Like apple juices, Esatbeyoglu et al. (2023) reported that the pH of *Aronia melanocarpa* juice fermented with kefir decreased.Bueno et al. (2021) stated that the pH values of red pitaya juices fermented with water kefir grains were reduced from 7.73 to 4.83. Similar patterns in TTA values have been observed by Randazzo et al. (2016); TTA increased after fermentation for apple, grape, pomegranate, and prickly pear, whereas it decreased for kiwifruit and quinince. This study observed the highest acidity in 48-h fermented kiwifruit juice with 18.69 g citric acid/L.

According to the studies of Silva et al. (2004) and Randazzo et al. (2016), the highest TTA observed in kiwi fermented juices was primarily due to malic and quinic acids, as well as citric acid. With longer fermentation times, fermentable sugars are metabolized more quickly, which reduces the amount of soluble solids (˚Brix) present (Paredes et al. 2022). There was an inversely proportional relationship between fermentation time and TSS in all fruit juices. Orange juice showed the least change in TSS amount with a 3% decrease in ˚Brix. There was a significant change in color parameters (a* and b*) by the fermentation time of apple, dragon, and kiwi fruit juices. This is in accordance with the results reported by Paredes et al. (2022). With respect to the control beverage, after 48 h of fermentation, redness (b*) values of the juices were significantly increased, except for orange juices. Randazzo et al. (2016), Corona et al. (2016), and Akarca and Baykal (2023) also reported that redness (b*) values significantly increased with fermentation, similar to our results. The ΔE value, which expresses the color change, was found to be the largest in the kiwi juice (8.26) and the smallest in orange juice (0.91) after 48 h of fermentation. The most noticeable changes were observed in apple and kiwi fruit juices. By considering the minimum observation threshold value of ΔE = 1.50 (Bi et al., 2020), all samples except orange juice color were changed the following fermentation. From the point of view, fermentation changes the color of the juices, similar to several reported studies (Corona et al. 2016; Randazzo et al. 2016; Wang et al. 2022). The bacteria and yeasts in the kefir culture may generate some organic acids that are relevant to the sensory quality of fermented beverages. All fruit juices contained malic, lactic, acetic, citric, fumaric, and ascorbic acids. Malic acid is the primary organic acid present in dragon and apple, as is also known from several studies (Betteridge et al. 2018; Bueno et al. 2021). After 48 h of fermentation of dragon juices, there was a significant four-fold increase in lactic acid (*p* < 0.05), additionally, acetic acid was also slightly increased after fermentation. Similar results were also reported for Tibetan kefir grain-fermented goji berry juice (Du et al. 2023) It’s likely that LAB consumption of sugars (hexoses and pentoses) might have caused the increased lactic acid content, which is an indicator of malolactic fermentation (Qin et al. 2018). Since water kefir microbiota consists of both homofermentative and heterofermentative lactic acid bacteria, organic acids such as acetic, formic, and citric acid are also formed, and these acids contribute to the formation of flavors and aroma of products (Ozcelik et al. 2021; Paredes et al. 2022). However, as shown in Table 3, fermentation had a negative impact on citric acid, which decreased from 29.35 g/L to 19.72 g/L in orange juices. A similar phenomenon has been observed in several studies (Paredes et al. 2022; Wang et al. 2022; Wu et al. 2020). Citric acid content decreased after fermentation due to LAB strains converted it to other metabolites such as diacetyl, lactic acid, and acetic acid (Wang et al. 2022). In addition to citric acid, lactic acid fermentation causes a reduction of malic acid content in juices, most likely owing to their role in the malolactic pathway and the tricarboxylic acid cycle (Li et al. 2021). Moreover, malic acid content was also decreased in orange and kiwi juices, and the decrease in malic acid level suggested that LAB used malic acid through the TCA cycle or malolactic fermentation (Chen et al. 2019). This was consistent with a previously reported finding that LAB utilised malic acid as a carbon source to produce lactic acid (Chen et al. 2019; Duan et al., 2023). Acetic acid concentrations were increased in all juices, except orange juice. From this viewpoint, acetic acid and lactic acid production are indicators of kefir microflora that use the heterofermentative route. Because the acetic acid production is not observed in orange juice, it can be assumed that the kefir microflora follows the homofermentative pathway (Du et al. 2023). The presence of small amounts of ethyl alcohol and in beverages like water kefir contributes the stimulating and yeasty aroma of the fermented finished products that are favorite by customers. According to the codex, non alcoholic beverages are characterized as beverages with ethanol content below 0.3% by volume in Non-alcoholic Beverages Notification of Turkish Food Codex (2007/26). In the majority of European countries, the threshold for a “no-alcohol beverage” is defined as 0.5% vol.; yet, a convincing justification for this level is sometimes missing (Okaru and Lachenmeier 2022). Accordingly, the higher viable counts of lactic acid bacteria in dragon and apple juices (Fig. 1) were in line with the sharp increase lactic acid content. TPC in fruits and vegetables are essential characteristics because of their good impact on several physiological processes connected to human health, such as cancer risk reduction, anti-inflammatory activities, and carcinogenesis inhibition (Wu et al. 2020). It has been reported by several researchers that TPC was increased during fermentation (Paredes et al. 2022; Zhao et al. 2023). The rise in overall phenolic content in juices during fermentation could be attributed to LAB hydrolases converting polyphenolic compounds to other compounds with lower molecular weight (Zhao et al. 2023). In line with our results, various researchers have reported reduction of TPC with fermentation (Corona et al. 2016; Esatbeyoglu et al. 2023; Randazzo et al. 2016; Wu et al. 2020). The breakdown of phenolic compounds occurs during fermentation, and the rate of this phenolic loss is responsible for the decrease in antioxidant activity. From these results, it might be claimed that fermentation time and conditions also affected the TPC. Significant differences were found between the TPC of apple, orange, and kiwi kefir-like beverages (*p* < 0.05). In the results of the DPPH assay, the change in antioxidant activity of dragon juice was not statistically significant (*p* > 0.05). Additionally, reductions in DPPH values with kefir fermentation have been reported in Mediterranean fruit juices (Randazzo et al. 2016), hawthorn, Cornelian cherry, rosehip, red plum, and pomegranate juice (Ozcelik et al. 2021), vegetable juices (Corona et al. 2016), red pitaya (Bueno et al. 2021), and a mixture of vegetable and fruit juices (Paredes et al. 2022). On the other hand, the DPPH content was increased only in fermented kiwi juices. One of the primary reasons for the strong antioxidant activity of water kefir is the presence of lactic acid bacteria in the kefir grain, as well as bioactive chemicals in the exopolysaccharide structure created during fermentation (Ozcelik et al. 2021). Furthermore, the higher antioxidant capacity by fermentation might be explained by the transfer of antioxidant compounds in kefir grains to beverages (Bueno et al. 2021). However, in the CUPRAC assay, a statistically significant reduction was observed in orange and dragon juices, whereas in apple and kiwi juices, a statistically significant increase was found. However, the impact of fermentation on the TPC and CUPRAC contents was different. In contrast, fermentation observed similar patterns in fruit and vegetable juices (Corona et al. 2016; Esatbeyoglu et al. 2023). The phenolic profiles of fermented fruit juices play an important role in determining their antioxidant activity and potential health benefits. The phenolic composition of fruit juices can indeed be affected by fermentation with water kefir. There were twelve phenolic compounds identified in orange juices. In the course of fermentation, the phenolic profile changes significantly (*p* < 0.05). Gallic acid, epicatechin and kaempferol were detected only in non-fermented juices. The presence of ferulic acid was not observed in non-fermented juices but appeared after 24 h of fermentation. During both 24 and 48 h fermentation, catechin, apigenin, rutin and quercetin-3-O-galactoside showed a decreasing trend. Fermentation did not affect hesperidin content. On the other hand, as fermentation progressed, chlorogenic acid and quercetin concentrations increased. Despite a decrease in caffeic acid content after 24 h, the level increased after 48 h of fermentation. Conversely, there was an increase in phlorizin content after 24 h, but a decline after 48 h.

There are several factors that can influence the changes in individual phenolic compounds during fermentation in fruit juices, including the type of fruit, the fermentation agent, the storage condition, and the processing method. A fermentation process can cause an increase or decrease in the levels of various phenolic compounds present in juices. A significant loss of phenolic compounds occurs during alcoholic fermentation in orange juice due to factors like precipitation, oxidation, adsorption with solids or proteins, and polymerization (Escudero-López et al. 2013). Similarly, the fermentation process with lactic acid bacteria in apple juice can lead to the degradation and oxidation of phenolic compounds, ultimately resulting in a decrease in their content (Wu et al., 2020). It is also important to note that different types of phenolic compounds are affected differently by fermentation. Furthermore, fermentation process can lead to the formation of new phenolic derivatives in the juices. In a study, fermentation of pomegranate juice with *Lactobacillus* strains resulted in the identification of new phenolic derivatives such as punicalagin and catechin which indicates the biotransformation of phenolics during fermentation (Valero-Cases et al. 2017). It illustrates the dynamic nature of phenolic compounds in fruit juices and their potential for transformation into novel bioactive components.

## Conclusion

In this study, probiotic beverage production by using water kefir grains with apple, dragon, kiwi and orange juices was performed. The lactic acid bacteria and yeasts exhibited higher than > 6 log counts in all fermented juices, except orange juice, which can be defined as being a probiotic beverage benefits. In terms of physicochemical properties, fermentation while pH was decreased, TTA was increased in all fruit juices. Moreover, at differerent fermentation period, physicochemical, functional and microbiological properties of the juices were examined. At the end of fermentation, the color parameters of the juices were significantly changed In contrast to kiwi juices, total phenolic contents and antioxidant capacity decreased after fermentation. Fermentation conditions also affect the functional characteristics and viability of probiotics. For this reason, optimization must be conducted during the production of functional products. These results provide valuable information about the development of innovative functional plant-based beverages for individuals with cow milk intolerances and those seeking nutritious meals with several health benefits. Considering this study, it can be inferred that by expanding the usage area of fruits to the production of functional and plant-based beverages. At the same time, today’s consumer demands are successfully met, fresh fruit losses can also be reduced by consuming fruits in different ways. The biochemical composition and physicochemical properties of these probiotic fruit beverages are well understood; however, the survival of lactic acid bacteria (LAB) and yeast in a simulated gastrointestinal tract, as well as sensory evaluation, require extensive investigation to achieve successful commercialization of novel fruit juice-based probiotic beverages.

## Electronic supplementary material

Below is the link to the electronic supplementary material.

Supplementary Material 1

## Data Availability

The authors confirm that the data supporting the findings of this study are available within the article. Raw data support the findings of this study are available from corresponding author, upon reasonable request.
