# Supplementary material for: Functional fermented fruit juice production and characterization by using water kefir grains
Source: J Food Sci Technol. 2025 Jan 29;63(2):378–91. doi: 10.1007/s13197-025-06209-y (PMC12926261; doi:10.1007/s13197-025-06209-y)
Supplement: Supplementary file 1 — Supplementary Material 1 [file 13197_2025_6209_MOESM1_ESM.docx]

**Supplemental File**

**Table S1.** Microbial loads of fruit juices fermented with water-kefir (log CFU/mL).

|  | Microbial Load (log CFU/mL) | | |
| --- | --- | --- | --- |
|  | ***Lactobacillus* spp.** | ***Lactococcus* spp.** | **Yeasts** |
| Fermentation at 0 h | | | |
| Apple Juice | 5.39±0.41^A^ | 3.22±0.22^B^ | 5.18±0.19^AB^ |
| Dragon Juice | 4.43±0.33^B^ | 3.30±0.16^B^ | 5.30±0.12^A^ |
| Kiwifruit Juice | 4.07±0.24^B^ | 4.11±0.08^A^ | 4.65±0.49^B^ |
| Orange Juice | 4.66±0.08^B^ | 4.29±0.44^A^ | 4.77±0.16^AB^ |
| Fermentation at 24 h | | | |
| Apple Juice | 6.42±0.16^A^ | 6.53±0.04^A^ | 6.74±0.06^A^ |
| Dragon Juice | 6.64±0.10^A^ | 4.20±0.25^B^ | 6.30±0.28^A^ |
| Kiwifruit Juice | 4.53±0.05^B^ | 6.18±0.02^A^ | 6.80±0.06^A^ |
| Orange Juice | 4.36±0.26^B^ | 4.37±0.06^B^ | 5.59±0.31^B^ |
| Fermentation at 48 h | | | |
| Apple Juice | 7.37±0.06^B^ | 7.23±0.05^A^ | 7.59±0.22^B^ |
| Dragon Juice | 7.78±0.04^A^ | 6.62±0.08^B^ | 8.57±0.18^A^ |
| Kiwifruit Juice | 6.15±0.04^C^ | 7.49±0.06^A^ | 7.12±0.10^B^ |
| Orange Juice | 4.34±0.15^D^ | 5.38±0.30^C^ | 7.61±0.39^B^ |

^A, B, C, D^ values within the same column at the same fermentation time with the different superscript upper case are significantly different (p<0.05).
